# “The support has been brilliant”: experiences of Aboriginal and Torres Strait Islander patients attending two high performing cancer services

**DOI:** 10.1186/s12913-021-06535-9

**Published:** 2021-05-24

**Authors:** Emma V. Taylor, Marilyn Lyford, Michele Holloway, Lorraine Parsons, Toni Mason, Sabe Sabesan, Sandra C. Thompson

**Affiliations:** 1 Western Australian Centre for Rural Health, The University of Western Australia, 167 Fitzgerald Street, Geraldton, Western Australia 6530 Australia; 2 Aboriginal Health Unit, Mission, St Vincent’s Hospital Melbourne, Fitzroy, Victoria Australia; 3 Townsville Cancer Centre, Townsville Hospital and Health Service, Douglas, Queensland Australia

**Keywords:** Aboriginal and Torres Strait islander, Indigenous Australians, Cancer services, Cancer care, Cultural safety

## Abstract

**Background:**

Improving health outcomes for Indigenous people by providing person-centred, culturally safe care is a crucial challenge for the health sector, both in Australia and internationally. Many cancer providers and support services are committed to providing high quality care, yet struggle with providing accessible, culturally safe cancer care to Indigenous Australians. Two Australian cancer services, one urban and one regional, were identified as particularly focused on providing culturally safe cancer care for Indigenous cancer patients and their families. The article explores the experiences of Indigenous cancer patients and their families within the cancer services and ascertains how their experiences of care matches with the cancer services’ strategies to improve care.

**Methods:**

Services were identified as part of a national study designed to identify and assess innovative services for Indigenous cancer patients and their families. Case studies were conducted with a small number of identified services. In-depth interviews were conducted with Indigenous people affected by cancer and hospital staff. The interviews from two services, which stood out as particularly high performing, were analysed through the lens of the patient experience.

**Results:**

Eight Indigenous people affected by cancer and 23 hospital staff (Indigenous and non-Indigenous) were interviewed. Three experiences were shared by the majority of Indigenous cancer patients and family members interviewed in this study: a positive experience while receiving treatment at the cancer service; a challenging time between receiving diagnosis and reaching the cancer centre; and the importance of family support, while acknowledging the burden on family and carers.

**Conclusions:**

This article is significant because it demonstrates that with a culturally appropriate and person-centred approach, involving patients, family members, Indigenous and non-Indigenous staff, it is possible for Indigenous people to have positive experiences of cancer care in mainstream, tertiary health services. If we are to improve health outcomes for Indigenous people it is vital more cancer services and hospitals follow the lead of these two services and make a sustained and ongoing commitment to strengthening the cultural safety of their service.

**Supplementary Information:**

The online version contains supplementary material available at 10.1186/s12913-021-06535-9.

## Background

Improving health outcomes for Indigenous people and reducing inequities is a vital challenge for the health sector worldwide [1, 2]. Cancer is a significant contributor to inequitable Indigenous health outcomes in Australia [3], as well as countries with similar histories of colonisation and marginalisation such as New Zealand, Canada, and the United States of America (USA) [4–7]. Cancer is the second most common cause of death for Aboriginal and Torres Strait Islander peoples [8, 9] who are significantly more likely to die from a diagnosed cancer than non-Indigenous Australians [10, 11]. (The term ‘Indigenous Australians’ is respectfully used hereafter to refer to Australia’s Aboriginal and Torres Strait Islander peoples.) Despite the significant improvements in cancer detection and treatments that have occurred over recent decades, Indigenous mortality rates from cancer are rising and the gap in cancer mortality rates is widening [11–13]. These poorer outcomes may be explained by a number of factors, including lower participation rates in screening programs, later diagnosis, lower uptake and completion of cancer treatment, the presence of other chronic disease, and systemic racism within the healthcare system [10, 11, 14, 15].

Studies have suggested a number of reasons why Indigenous Australians might not wish to attend cancer services, including fear or lack of trust of mainstream health facilities, lack of understanding or respect shown by health care providers, experiences of racism, fatalistic or differing cultural beliefs about cancer, including feelings of shame, and logistical difficulties in accessing screening and treatment services [16–19]. The accessibility and cultural safety of a health service has a significant impact on whether Indigenous Australians are willing to present for diagnosis and continue with treatment [15, 20, 21]. The concept of ‘cultural safety’ was developed in Aotearoa/New Zealand to respect Maori culture and was originally defined by Ramsden as *“questioning power relations between [the] nurse and the person being nursed with the emphasis on the attitudes and behaviours of the nurse … That is, the enactment of Cultural Safety is about the nurse while, for the consumer, Cultural Safety is a mechanism which allows the recipient of care to say whether or not the service is safe for them to approach and use”* [22]. In Australia, the Australian Health Practitioner Regulation Agency (AHPRA) states that “*cultural safety is determined by Aboriginal and Torres Strait Islander individuals, families and communities*” and defines culturally safe practice as the “*ongoing critical reflection of health practitioner knowledge, skills, attitudes, practising behaviours and power differentials in delivering safe, accessible and responsive healthcare free of racism*” [23].

Attention must therefore focus on how cancer services can provide culturally safe care in order to encourage Indigenous Australians to attend cancer services and improve outcomes for Indigenous people with cancer. This includes providing person-centred care that meets the varied needs of Indigenous Australians including the physical, psychological, social, cultural and spiritual aspects of their health [24]. Recent publications including the *National Aboriginal and Torres Strait Islander Cancer Framework* [25], *The Optimal Care Pathway for Aboriginal and Torres Strait Islander people with cancer* [26] and the *Australian National Safety and Quality Health Service (NSQHS) Standards User guide for Aboriginal and Torres Strait Islander health* [27] emphasise the importance of culturally safe, person-centred care and outline a number of areas to be considered when supporting the delivery of optimal care to Indigenous people. Despite these publications, many cancer providers and support services while committed to providing high quality care, often struggle with providing accessible, culturally safe cancer care to Indigenous Australians [24, 28, 29]. In the past decade, reports have begun to emerge of cancer services that provide innovative services to engage Indigenous patients and their families [30–33] although limited formal evaluation of successful cancer service delivery initiatives for Indigenous Australians have been reported to date. It is essential that any attempt to evaluate such cancer service initiatives include the perspectives and experiences of Indigenous Australians as a core component.

Until recently limited information existed on where Indigenous Australians receive cancer treatment [29, 34] and there are few reports in the literature of Indigenous Australians having positive cancer care experiences [30, 35]. To find out where Indigenous Australians received cancer treatment, a survey of public cancer treatment centres across Australia was undertaken to identify the type of cancer services provided, collect Indigenous patient numbers and explore policies and implementation of Indigenous-specific initiatives [36]. Surveys were completed for 58 of the 125 public cancer treatment centres. Results of the survey led to further refinement and follow-up interviews with service providers to explore current practice and programs towards improving cancer care for Indigenous Australians [37]. Two cancer services within large hospitals were identified as particularly focused on their performance in providing cancer care for Indigenous cancer patients and their families. The aim of this study was to explore the experiences of Indigenous cancer patients and their families within the cancer services and ascertain how their experiences of care matched with the cancer services’ strategies to improve care.

## Methods

### Study design, service selection and profile

This study forms part of a national project to identify and describe cancer services providing treatment to Indigenous cancer patients in Australia. Case study methodology “*the intrinsic study of a valued particular*” [38] was used because of its particular relevance to this component of the project, with our cases selected “*not because [they are] representative of other cases, but because of [their] uniqueness, which is of genuine interest*” [39]. Furthermore, case studies can provide value in Indigenous research if done sensitively and appropriately [40, 41].

Initially, six centres which had reported promising practices were identified, with five agreeing to participate in more detailed study around their specific practice and innovation. One centre has previously been reported [42] and another is in the process of data collection and analysis. Unfortunately, changes which had occurred within one of the identified services meant it was no longer considered by the research team to be high performing with respect to specific attention on care of Indigenous people; therefore no information from this service was included in the final analysis. Based upon interviews and observations, the two health services described here were considered high performing and innovative in their provision of cancer services for Indigenous cancer patients and their families. An article describing the inclusive and supportive Indigenous workforce policies and strategies of the two services has recently been published [43]. This article concluded that positive patient outcomes and a strong Indigenous health workforce can be achieved when a health service has strong leadership, commits to an inclusive and enabling culture, facilitates two-way learning and develops specific support structures appropriate for Indigenous staff [43].

The selected services represent some of the diversity that exists between health services in Australia. The cancer services are both located within public tertiary teaching hospitals, however differ with respect to rurality, management and patient cohort. The Urban Service is located in a major capital city, within a privately run hospital with nearly 900 beds, and employs more than 5700 staff (0.9% identify as Indigenous) in a state where Indigenous Australians account for 0.8% of the state’s population. The Regional Service is located in a large regional centre over 1000 km away from the next tertiary hospital, is a public hospital run by the state health service, has almost 800 beds and employs more than 6000 staff (3.7% identify as Indigenous); with Indigenous Australians accounting for approximately 8% of the region’s population.

### Cultural and ethical considerations

Ethics approvals for the national study were provided by the Western Australian Aboriginal Health Ethics Committee (WAAHEC) (approval number 483) and the Human Research Ethics Committee of University of Western Australia (RA/4/1/6286), with overarching ethics approval for the specific sites provided by St Vincent’s Hospital Melbourne Human Research Ethics Committee (approval number HREC/16/SVHM/94).

The research project was embedded in the Centre for Research Excellence (CRE), Discovering Indigenous Strategies to improve Cancer Outcomes Via Engagement, Research Translation and Training Centre of Research Excellence (DISCOVER-TT). The CRE was led by an Indigenous researcher, and brought together Indigenous and non-Indigenous researchers, service providers, policy-makers and consumer groups, with the aim of improving outcomes and services for Indigenous people with cancer.

We adhered to the National Health and Medical Research Council (NHMRC) Guidelines for Ethical Conduct in Aboriginal and Torres Strait Islander Health Research [44]. Central to our research were the values of reciprocity, respect, equality, responsibility, survival, protection, spirit and integrity, which guided study design and conduct. Three Aboriginal women were members of the research team, with roles that included input into the study and data collection, undertaking interviews, and input into and approval of the draft manuscript. An Indigenous Advisory/Reference Group was formed prior to commencing the case studies. This group had face-to-face meetings and provided advice and support to the study. In addition, we consulted with local Aboriginal Community Controlled Health Organisations (ACCHOs).

### Participant recruitment and data collection

Adults aged 18 years and over were eligible if they were Indigenous, affected by cancer (Indigenous person diagnosed with cancer or a family member) and had experienced or observed cancer care at one of the two sites in this case study, or health professionals and support staff (Indigenous or non-Indigenous) involved with the care or support of Indigenous cancer patients or who filled a leadership role in the care of Indigenous patients.

Recruitment was purposive, with Indigenous cancer patients and family members and relevant staff identified and recruited in-person by local health service staff and managers within each participating organisation. A snowball-sampling recruitment strategy was also followed with staff, whereby participating staff could identify additional potential staff participants who they felt would provide additional valuable insights. The site investigator ensured that all potential participants were informed and prepared prior to being approached by researchers for interview. Interview participation was voluntary. All participants gave written consent prior to data collection and were reminded that they could stop the interview at any time. Indigenous patients were encouraged to have a family member or support person present during the interview if they wished.

Semi-structured interviews were conducted with Indigenous people affected by cancer (*N* = 8) and hospital staff (*N* = 23) from the two services. Interviews took place between September and December 2017 at the Urban Site and between November and December 2018 at the Regional site. Two interview guides were developed by the research team, one for Indigenous people affected by cancer and one for health professionals. The interview schedules guided the direction of the interviews; however, individual circumstances occasionally resulted in the inclusion of additional questions. Interviews ranged from 30 to 60 min in length and were conducted with the use of the appropriate interview guide, audio taped with consent, transcribed verbatim and de-identified prior to analysis. The interview team consisted of four women (ML, LP, SCT and a Research Assistant). Two of the researchers conducting interviews were Aboriginal women, both of whom have clinical backgrounds in cancer, and one of whom is an experienced researcher. The two non-Indigenous interviewers both have clinical backgrounds and over twenty years’ experience with collaborative research into improving Indigenous health outcomes. Most interviews were conducted jointly by two interviewers who debriefed and discussed observations after each interview.

An Indigenous researcher participated in all interviews with Indigenous patients and family members, all of which were conducted in person. Interviews were undertaken at a convenient time and location for participants, usually at the health service. Two patients chose to have a family member present when interviewed and, on two occasions, two staff members chose to be interviewed together. Indigenous patients with cancer and family members were asked about their experiences at the cancer centre and any suggestions for changes or improvements. The interview guide for people affected by cancer is presented in Additional file 1. Health professionals were guided through a range of enquiries pertaining to initiatives and programs undertaken to improve engagement with Indigenous people, with particular emphasis on cancer patients, including questions on cultural awareness programs, cultural identifiers and Indigenous staff employment strategies and numbers. The interview guide for health professionals is presented in Additional file 2.

### Data analysis

We followed the qualitative data analysis method described by Green et al. [45] of immersion in the data with rereading, coding, categorisation and aggregation of identified themes. Preliminary coding was carried out by an independent qualitative coder using NVivo 10 to organise and extract relevant data and identify themes. Discussions within the team were undertaken to refine themes and triangulate patient and provider interviews.

All patient and family member interviews were then re-read and manually coded by a team member (EVT) to ensure that the patient experience was fully captured and to develop existing themes and identify additional patient-centric themes. Data from the cancer services was then mapped to themes identified in the patient data, to see whether and how the cancer services strategies to improve care matched the patients’ experiences. Including the views of Indigenous patients, family members and hospital staff allowed for a triangulation of perspectives, and provided a contextualised understanding of how cancer care is both experienced by and provided for Indigenous people, with differing perspectives being identified and explored. Provisional data interpretation was checked with key stakeholders at each service, with feedback and additional information incorporated into the final analysis. Finally, the team met to further refine themes and reach agreement on the final themes.

## Results

### Participant characteristics

Almost half (*n* = 15) of the 31 participants identified as Aboriginal or Torres Strait Islander (Table 1). Eight Indigenous people affected by cancer were interviewed, of which five were Indigenous patients with cancer (four male) and three were affected family members (all female). Although participants were split evenly between the Urban and Regional Services, most (*n* = 7, 87%) lived in a regional or remote area.

**Table 1:** Characteristics of study participants

**Indigenous people affected by cancer (*n*** **= 8)**			
	**Patients**	**Family members**	**Total**
	5	3	8
Gender			
Female	1	3	4
Male	4	0	4
Service			
Urban Service	3	1	4
Regional Service	2	2	4
Place of residence			
Urban	1	0	1
Regional	3	3	6
Remote	1	0	1
**Health Service Staff (*n*** **= 23)**			
	**Urban Service**	**Regional Service**	**Total**
	10	13	23
Gender			
Female	8	11	19
Male	2	2	4
Indigeneity			
Indigenous	3	4	7
Non-Indigenous	7	9	16

Of the 23 hospital staff, 13 participants were employed at the Regional Service and ten participants worked at the Urban Service. Staff included more women (*n* = 19, 83%) than men, and almost a third (n = 7) identified as Aboriginal or Torres Strait Islander. Participants included Indigenous Liaison Officers (ILOs) [titled Aboriginal Liaison Officers (ALOs) in some states of Australia], oncologists, registered nurses (RNs), social workers, managers, executives and administrative staff.

Analysis of the interviews revealed three experiences that were shared by the majority of Indigenous cancer patients and family members interviewed in this study: a positive experience while receiving treatment at the cancer service, a challenging journey to the cancer service and the importance of family support. Participants also made a number of suggestions for improvements to the health system which could be grouped into three broad categories: provide additional funding for Indigenous people affected by cancer and cancer services; increase the amount of information on cancer available to the Indigenous community; and show more compassion and understanding towards Indigenous people.

### “They have gone above and beyond”: positive experience of Cancer care

#### Patients’ perspectives

All patients and family members at both services spoke positively about their experience of cancer care referring to the support, treatment and communication they received from the staff within the cancer services, and highlighting the importance of the ILOs. There was the sense that while getting to the cancer service may have been challenging, once they arrived things were easier and, in the words of one patient, *“straightforward after that”* (Patient 2).

Participants emphasised the importance of the support that they’d received from the cancer service staff. In the words of one participant: *“Yeah, the support has been brilliant. I can’t thank you enough for the support you have given me and my family. To me, that helps me more than the chemo”* (Patient 1). Even when not actively receiving care, patients and family members felt comfortable contacting staff at the cancer service for support: *“Every time we’ve needed them, all we have had to do is just ring them and they would offer advice over the phone or what to do”* (Carer 1). Individual staff members were mentioned as providing particular care or going *“above and beyond”* (Patient 3). One patient described the Nurse Unit Manager (NUM) of the oncology ward as *“my guardian angel”* because of the support she provided by staying with the patient *“when the doctors came around, she stayed because my daughter and [husband] weren’t around, and I think that is lovely”* (Patient 5). Participants also praised the quality of their treatment. *“From the first time that I went in to the time that I left I received the best treatment that anybody could have received”* (Patient 2).

In contrast with reports of poor communication before reaching the cancer service, participants spoke positively about the amount of information they received and how well everything was explained to them and their family members at both cancer services. One patient at the Regional Service described how staff at the cancer service contacted him prior to his relocating for treatment, and how their explanations helped him to prepare: “*They [the staff] explained everything. They made sure I knew everything before, you know, like they started everything. I knew where to go and how to go about it and what to do”* (Patient 4). A patient at the Urban Service spoke in glowing terms about information provided to him and his family:

*The information that they have given me about the different treatments and the treatment that I’m getting has been phenomenal. Not only has one of the nurses even sat down with me with the information and gone through it, but also the doctors have … . Yeah, and also my family too. They have also received the information that they needed to receive as well.* (Patient 1)

Participants stressed the importance of the support provided by the ILOs who worked within the Cancer Service at both services. Several patients described how the ILOs increased their comfort within the service through their presence and interactions.

*They send different people to you, like oncology psychologists and things like that, but I didn’t really want to open up to them, do you know what I mean? But, as I said before, once again I had [ILOs], another bloke over at the Aboriginal liaison, so they were handy to talk to. (Patient 3)*

One patient described how the support from the ILO helped them to stay in hospital (and not discharge against medical advice):

*I have really appreciated the support I’ve received from the Aboriginal liaison officer … they have gone above and beyond what they have to do just to make sure I’m right. Without their support I don’t know where and what headspace I would be in. I probably wouldn’t even still be in hospital. They have done all they can to keep me here and, yeah, I probably would have done a runner and gone back to [home town] by now if it wasn’t for their support and understanding.* (Patient 3)

This support was especially important for rural residents relocating to the Regional or Urban Service for treatment, given the unfamiliar surroundings and lack of family support. Most regional patients who had to relocate for treatment mentioned the logistical support provided by ILOs, such as help organising transport and accommodation. A regional patient described how the ILO helped them when they first arrived: “*Yeah, [the ILO] helped us out a lot here too …*. *they helped me out with food vouchers and all that sort of stuff when I first came down because we had no money, you know*” (Patient 4).

#### Service providers’ perspectives

All staff members interviewed talked about the importance of providing quality care for Indigenous people both at their health service and as a personal ethos. Staff mentioned using a range of approaches to provide safe and supportive care to Indigenous patients including: spending extra time with them, making sure they felt safe, involving them in the decision making process, providing services via telehealth, and being mindful of *“specific cultural or spiritual requirements or beliefs*” (Participant 6, NUM, non-Indigenous). The collective sentiment can be summarised in the following statement by a Cancer Nurse Consultant: “*that is really what is important to us, ensuring that our [Indigenous] patients feel safe, that they feel like they are getting high quality care, they can ask us for help at any time and that we are there for them*” (Participant 5, Nurse, non-Indigenous).

Staff at both services recognised that Indigenous patients from regional or remote communities required additional support and understanding:

*They are often far away from home, away from Country, away from family, and in a hospital environment which is such a complex place to be for many Aboriginal people, too, so it is around how can we enhance communication and how can we make them feel more safe and more comfortable*. (Participant 2, Social Worker, non-Indigenous)

Oncologists at the Regional Service talked about the importance of understanding if the patient was using bush medicine, and approaching that in a respectful, inclusive and non-judgmental way. Patients were asked *“Please let me know because we want the benefit of both of these things. We don’t want them to work against each other, so we just need to know.*” (Participant 14, Oncologist, non-Indigenous) On several occasions the bush medicine practitioner was included in consultations.

All staff stressed the importance of involving and working with the ILOs when caring for Indigenous patients. ILOs were valued members of the multidisciplinary team, attending regular clinical meetings and speaking on behalf of Indigenous patients. ILOs fulfilled an important navigator or care coordinator role, as well as providing more holistic support. Staff at both services mentioned the *“automatic”* involvement of the ILO with any Indigenous patient, usually on the patient’s first visit, and a co-working model. This model was formalised at the Urban Service, where joint patient assessments conducted by a Social Worker and an ILO were *“expected”*. Social Workers perceived benefits such as improved cultural safety for the patient, having a respected *“cultural expert”* to help them advocate for the patient and enhanced communication with the patient. In the Regional Service, both Indigenous and non-Indigenous staff talked about the importance of working together when consulting with Indigenous patients to improve communication and cultural safety.

*I will often have an Aboriginal liaison officer with me. I will try and just understand a little bit before the time from the Aboriginal liaison officer about this particular person’s position in the community, what is acceptable in terms of direct eye contact or not, just the kinds of thing like that, just to try and demonstrate a sense of respect.* (Participant 14, Oncologist, non-Indigenous)

While palliative care was not mentioned by Indigenous patients or family members, staff at both services recognised the importance that many Indigenous patients placed on going home to die – dying on Country. Nurses and ILOs described going *“above and beyond to try and achieve that [patient returning home to die]”* (Participant 6, Nurse Unit Manager, non-Indigenous). *“A lot of our mob like to go home to Country to die, so we will go into meetings and advocate for them to go home”* (Participant 17, ILO, Indigenous). Staff followed the lead from ILOs unless end-of-life wishes were clearly communicated by the patient and his/her family members. Nurses, doctors and social workers prepared and assisted patients with Advanced Care Directives (ACD), which were important when dealing with a family who were undecided on a course of action for a dying relative. However, staff at both services acknowledged misunderstanding in the community about the nature of palliative care, including the misconception that it meant death was imminent, which made conversations about palliative care challenging. In these cases, the importance of ILO involvement and having conversations early was emphasised. Early engagement in end-of-life planning with Indigenous palliative patients also enabled timely referrals from palliative care services to community service providers when a home transfer was requested.

Both services had a culture of continuous improvement and were actively and continually trying to improve outcomes for Indigenous patients and strengthen the cultural safety of their services. Staff at the Urban Service described how the ILO would routinely contact Indigenous patients after discharge, and ask for feedback on the service including questions on support provided, contact for follow-up appointments, communication, and how the service could be improved.

The cancer services in this study do not exist in a vacuum and their organisational culture and the way they provide services is directed and influenced by the hospitals they are part of. Both the Urban and Regional health services in this study demonstrated a strong and positive commitment to improving Indigenous patient care. This is evidenced in both services through: improving Indigenous health outcomes being a key commitment of their strategic plans; the existence and continued development of a Reconciliation Action Plan (RAP); the establishment of Indigenous Health Units, with an emphasis on Indigenous employment across all departments; and mandatory cultural awareness training for all staff. The Regional Service has Indigenous leadership at the highest levels, including an Executive Director of Aboriginal and Torres Strait Islander Health. Comments from multiple participants at the Urban Service showed that Executive support in this area was felt by staff on the ground, as epitomised by the comment *“We certainly do have the support of executive members to do what we need to do to have good outcomes”* (Participant 7, Manager, Indigenous).

### “Getting here”: journey to cancer service is challenging

#### Patients’ perspectives

Three-quarters of the Indigenous people affected by cancer in this study (*n* = 6) reported difficulties before reaching the cancer service. While not an intended focus of the study, and not explicitly enquired about, participants reported that the time between them (or their relative) first experiencing symptoms and reaching either the Urban or Regional Cancer Service was *“confusing”*, *“stressful”*, and marked by medical delays, misdiagnosis and poor communication from health service providers. Some participants also reported reluctance to leave home and travel to the city for treatment. Negative experiences were reported with local health clinics, other hospitals and with non-cancer wards within the two health services in this study.

Participants experienced a number of delays with diagnosis, including delays caused by misdiagnosis. In local health clinics this was exacerbated by the patient being seen by multiple doctors or doctors who were not familiar with the patient, resulting in poor continuity of care and slower follow up of results. In one case, diagnosis and hospital admission was also delayed by slow communication from a major public hospital, and in the end the participant was admitted, had surgery, and had been receiving ongoing treatment from the Urban Service for over a month before the letter came from the other public hospital telling them to come for their first visit.

*My doctors at the local clinic, because it is one of those community clinics where there are a number of doctors, I had three different doctors looking at this, and it was only the last one who said that this couldn’t keep going on because it had taken well over a month to get anything back, any reports back that were of any value [from major public hospital] ….* (Patient 2)

*So we went to a clinic in [regional centre]. You go through the tests... I had chest X-rays, ultrasounds and all this stuff, and eventually after I think two weeks she told me I had glandular fever. By that time I had pain in my left side... And pain, you ask me out of 10 and I’ll tell you 12 … I cried to my husband. I said, ‘I can’t do this anymore.’ I said, ‘I just want to die’ … I was really crook … And so I went to ED [Emergency Department]. I went another time and they couldn’t find anything. I thought, ‘I must be okay’. But that last time I went to ED, they said, ‘Oh no, I think we’ll keep her in for a couple of days.’ And I thought, ‘Okay, that’s a start’. You know, I’ll get some help.* (Patient 5, who was later diagnosed with a rare lymphoma)

Once diagnosed, participants reported a range of difficulties including receiving misleading information from their local doctor about how long they would be required to relocate for tests or treatment, not knowing where to go once they’d reached the tertiary health service, lack of communication from health service providers about their diagnosis and treatment, and poor treatment from health providers at a different hospital. One family member talked about how the oncologists at their local hospital *“just didn’t seem to care”* about her brother and “*sent him home to die”* (Carer 1), which resulted in her brother seeking treatment from the Urban Service. Several participants talked about a lack of communication from health service providers, or communication being provided at inappropriate times or being hard to understand and *“going over their head”* (Patient 3).

*If there is one thing that was confusing for me was my initial entry into the hospital … I had a lump in my throat which was removed and I was kind of given a very quick response to that. Like, it wasn’t explained a lot, and I think that when it was explained to me I was still coming out from the anaesthetic, and the next time I had someone speak to me it was all very quick and it was like I understood all the medical jargon and medical terms … that was probably the only time that I felt uncomfortable possibly with not knowing what was going on. I had a lot of people who were very anxious about me and about the treatment, about the diagnosis, and yet it was very difficult to find someone to actually tell me anything about my particular case.* (Patient 2)

Although not generalisable, two participants spoke about feeling that some health service staff made assumptions about them and that they received poor treatment before reaching the cancer service, which they felt was due to their race.

*When I first checked in they had me prescribed for Valium because they thought I would be withdrawing from alcohol because they already had an idea that I would be an alcoholic just because I was Aboriginal.* (Patient 3)

*I did ask one of the nurses if I could have a shower because I had rashes all here all on my back, and I was hot …. They said, ‘We haven’t got time’... I did ask one of the staff that night if I could have a fan. They gave me a strip off a box. He gave me a strip off a box …. I was fanning myself, and to me that was neglect.* (Patient 5)

#### Service providers’ perspectives

Although the issues described above occurred before the patients reached the cancer service, and were therefore mostly outside of their control, staff at both services were aware that for Indigenous patients simply *“getting here”* was a barrier. As a NUM of the oncology ward explains, *“By ‘getting here’, I don’t mean transport. I mean, you know, mentally getting into the hospital system and that. I think that that is a barrier”* (Participant 1, NUM, non-Indigenous). Staff acknowledged that the hospital system could be *“overwhelming”* and *“disempowering”*, particularly for patients who had to relocate for treatment.

Both services in this study had implemented a number of strategies in an attempt to mitigate the barriers experienced by patients before commencing cancer treatment. Notably, the Urban Service had a program whereby patients could be admitted directly to the oncology ward and bypass the Emergency Department. Although this initiative was for all cancer patients, it was believed that it would particularly benefit Indigenous patients by reducing the stress associated with Emergency Departments and potential lengthy waiting times. The service also attempted to provide a *“one-stop shop”* for regional patients *“so they have their consult the same day as their treatment … so we try and do everything so they don’t have to come back twice”* (Participant 1, NUM, non-Indigenous).

In addition, a number of staff talked about spending extra time and providing additional information to Indigenous patients prior to them commencing treatment. For example, an ILO at the Regional Service described how additional time was spent educating Indigenous patients on their treatment to help them feel more comfortable:

*And then the treatment part is discussed with the patient, you know, radiation or chemo. They don’t know what you are talking about. So, you know, we take them around and we show them. They are very good here. They do it with every patient, but with our [Indigenous] patients, you know, extra time is needed because they are scared, you know. We show them the difference between radiation and chemo.* (Participant 17, ILO, Indigenous)

Staff at both services also talked about the importance of involving the ILOs as early as possible to help safely transition Indigenous patients into the cancer service. In the case of the Regional Service, it was described how the ILO usually made contact with patients before they started treatment to help them “*feel more comfortable coming”*, and how the ILO would meet patients on their first visit *“and made them feel comfortable and showed them around, you know, prior to their actual treatment*” (Participant 22, Admin, Indigenous).

### “Your family is number one”: importance of family

#### Patients’ perspectives

Three-quarters of the Indigenous people affected by cancer in this study (*n* = 6) stressed the importance of family support, which started with receiving their diagnosis, through to relocating for tests and treatment, and while receiving treatment. The presence of family helped with patients’ mental health and aided communication with health professionals. Family members also assisted with transport and accommodation, particularly when patients had to relocate or travel long distances for treatment. However, patients acknowledged a considerable burden on the family members responsible for caring for them.

Two regional patients talked about how their mental health was improved because of the presence of their family during treatment. One patient described how his partner helped keep him in hospital receiving treatment: *“I don’t know if I would still be here if I was on my own. Like, she [partner] sort of picks me up on the way downhill, you know”* (Patient 4). Another patient talked about how the presence of their family helped them to come to terms with their cancer diagnosis:

*In the beginning I felt ‘Why?’ Like, ‘I can’t believe that I’ve got cancer’. Yeah, that sort of thing, but that was really quick and brief because I had my family here with me, which is how I know it makes everything just so much easier.* (Patient 1)

Two patients talked about how having a family member present helped improve communication with health professionals. One patient stressed that it wasn’t the support so much as “*another pair of ears that understands”* (Patient 2), whereas another patient needed his partner because “*she knows more about these big words than what I know”* (Patient 4).

However, most patients acknowledged the burden on their carers – both from a time and financial perspective, and stressed the need for more funding to support carers. The burden on carers was exacerbated when regional patients had to relocate or travel for treatment. Patients and carers described regularly driving long distances to transport patients, or needing to drive patients around unfamiliar cities. Furthermore, they reported a significant financial burden on the carers who had to relocate to support the patient. A regional patient described the difficulties experienced by his parents when they relocated to the city to help care for him during his treatment: “*It is a struggle on them obviously with costs of living and food, and coming from the country to the city is another challenge in itself for them too*” (Patient 1). One carer described giving up her job and relocating to a remote community to support her parents after first one, and then the other, was diagnosed with cancer. The large size and importance of extended family for many Indigenous people is not accommodated by funding schemes that only provide support for one carer to accompany a patient when they relocate for treatment. One patient reported this was insufficient because, due to their own health conditions, both his parents had to relocate to support him during his treatment.

*Your family is number one. Like, when it comes to these sorts of things you want … not just one person, but usually you want your immediate family there with you, so that is like two or three or four people …. And aunties and uncles, and they all want to be here … but it just makes it easier if there was not just funding for one but maybe two, you know, mum and dad, for example.* (Patient 1)

Large family size and the extended kinship network was beneficial when caring responsibilities could be shared between several family members, however, in several cases resulted in one family member caring for multiple relatives. One patient describes how her daughter ended up supporting multiple family members and acquaintances who were all receiving treatment at the Regional Service:

*She [daughter] has not only helped helping us [both parents], but she has helped [names three other Indigenous patients], and then our nephew, his daughter came in on a chopper and he was all upset sort of thing. Anyway, she told us, ‘Take him back to the unit there’ and then she was mentoring him.* (Patient 5)

#### Service providers’ perspectives

Staff at both services were aware of the importance of family for Indigenous patients and worked to accommodate the needs of the patient and their family within existing system barriers. The range of ways to support families of patients included providing sufficient physical space for the whole family to gather, being flexible with methods of communication including using telehealth as a means of communication to enable all family members to be involved, and providing logistical support to family members such as “*the occasional meal for mum and dad, parking, for instance, because where they are staying has no parking, so the car is here at the hospital*” (Participant 4, ILO, Indigenous).

One staff member at the Regional Service talked about the difficulties created by the limited size of many hospital rooms, and how they tried to accommodate large families so that they felt comfortable:

*We try and find a space [for family]. Unfortunately, the hospitals are not well designed and there is limited space. Usually we try and find an area or a space that they might want to be in, and there is a little room at the end. It is a family room with a TV, so often if there are lots of people … We’ve had like 15 people in a room actually, but we will always say, ‘Are you comfortable here? Do you want to go somewhere else?’ They try and accommodate families as much as they can, yeah, realising that family is extremely important, yeah.* (Participant 20, Social Worker, non-Indigenous)

Staff at both services talked about the importance of making sure all family members were kept updated. A Social Worker at the Urban Service described first checking with the patient about who they would like involved and stated that “*even if it is a list a mile long that is fine … we can make more than one call if we need to or we can advocate that a family meeting or something might be better to get everyone in to have those frank discussions*” (Participant 2, Social Worker, non-Indigenous). At the Regional Service, where there were often great distances separating patients from families, staff used telehealth as a means of communicating with families and keeping families involved with patients’ care.

*The video is powerful to actually see it, because our mob, we are touch, feel, smell, see, so for them to actually see a video and to see that their uncle, their nephew, their brother, on this bed in so much pain, the reality has actually hit back home, like, ‘We need to come down and see him. We need to visit him. We need to make contact with him’.* (Participant 16, ILO, Indigenous)

## Discussion

This study captures the views of Indigenous people affected by cancer regarding their experience of care at two cancer services. In addition, it reports the views of health professionals working at both services. Three experiences were shared by the majority of Indigenous cancer patients and family members interviewed in this study: a positive experience while receiving treatment at the cancer service; a challenging time between receiving diagnosis and reaching the cancer centre; and the importance of family support, while acknowledging the burden on family and carers. While acknowledging the limitations of the small sample size in these two service case studies, the positive care experiences reported by patients and family in this study reinforce previous findings that these services are performing well in their provision of cancer services for Indigenous peoples [37, 43]. This study suggests that the respectful, person-centred culture of these two services, as well as the supportive strategies implemented, are meeting the needs of at least some of their Indigenous cancer patients. The findings also suggest that, with a coordinated person-centred approach which involves patients, family members, Indigenous and non-Indigenous staff, Indigenous people can have positive experiences of cancer care in mainstream, tertiary health services, something that has rarely been reported in the literature. However, even within these exemplary services, gaps exist between the services they provide and the wider healthcare system, with more support needed to help patients reach the cancer service in the first place and more support needed for families and carers of patients.

This is one of the first reports in Australia where all Indigenous participants spoke positively about their experience of cancer care. This contrasts with the literature, where despite the publication of the *National Aboriginal and Torres Strait Islander Cancer Framework* [25], the *Optimal Care Pathway for Aboriginal and Torres Strait Islander People with Cancer* [26] and the *Australian National Safety and Quality Health Service (NSQHS) Standards User guide for Aboriginal and Torres Strait Islander health* [27], reports of Indigenous Australians’ having positive cancer care or tertiary health care experiences are rare. However, it should be recognised that identifying the two services that participated in this study was a long and considered process, involving a national survey, followed by interviews with many services, with these two services invited to participate in a case study because they appeared to be particularly high performing in their provision of cancer services for Indigenous peoples. Furthermore, the cancer services in this study are directed and influenced by the hospitals they are attached to. Both the Urban and Regional health services in this study have made a long-term and continuing commitment to improving Indigenous patient care, with strong leadership from their executive teams, measurable targets to improve Indigenous health in their strategic plans, policies and ongoing efforts to develop a supportive work environment and respectful culture for both Indigenous staff and patients [43].

A key component to participants’ satisfaction with the cancer services was the high level of communication they received from staff. This contrasts with some participants’ experiences of poor communication from health care providers prior to reaching the cancer services and with the literature, which is full of reports of poor communication by health service providers [46–48]. However, effective communication has repeatedly been found to be a critical component to improving Indigenous engagement with healthcare services [49–51], with patients who received timely and effective communication about their cancer and treatment reporting a more positive experience of care [46]. The *Optimal Care Pathway for Aboriginal and Torres Strait Islander People with Cancer* states that a fundamental step to improving health outcomes is culturally respectful communication, and it makes a number of suggestions as to how this can be achieved, such as *“making time during explanations and appointments to build rapport and trust”*, asking one question at a time and asking the patient whether they would like an Indigenous Health Worker (IHW), ILO or family member present [26]. The positive communication skills demonstrated by staff at these services could have been developed through attending the mandated cultural awareness training, which may help to improve cultural competence and cross-cultural communication skills [27, 52]. However, it should be acknowledged that cultural awareness training, especially when it is done to ‘tick a box’ and without involvement of local Indigenous communities, does not automatically lead to culturally safer organisation [53]. More powerful than any mere cultural ‘awareness’ or ‘competence’ was the deep respect for Australia’s Indigenous Peoples that came through strongly in every staff interview, as well as the ethos that that *“what we do [in Indigenous health] is Aboriginal led”* (Participant 10, Project Officer, non-Indigenous).

The *Optimal Care Pathway for Aboriginal and Torres Strait Islander People with Cancer* suggests using telehealth to help facilitate effective communication, as well as a means to improve access to specialist services [26]. Telehealth has previously been found to be an acceptable and effective communication tool by Indigenous patients and their families [30, 54], as well as improving social and emotional wellbeing, access to health services and clinical outcomes [55]. Telehealth has increasingly been adopted as an important element of regional and remote health service provision, the use of which has dramatically increased during the COVID-19 pandemic [56]. Concern over the impact of distance and travel on Indigenous and other rural patients led to the development of a telehealth system at the Regional Service. Telehealth services are now used to provide telehealth-supported chemotherapy services at select remote satellite sites and follow-up consultations with the oncologists. This reduced the number of visits to the Regional Service and enabled some rural/remote patients to remain closer to home for ongoing treatment. Patient benefits included reduced travel costs, reduced waiting time, inclusion of family in consultations and treatment plan discussions, and reduced stress caused by travel to an unfamiliar city and the challenging hospital environment [30].

Another key factor in participants’ satisfaction with the services was having the ILOs highly involved in their care. The numerous benefits of ILO involvement have been reported previously [57–59], with the Australian Health Practitioner Regulation Agency (AHPRA) acknowledging that better patient outcomes are more likely if one of the attending health professionals is Indigenous [60]. The services in this study comply with the *Optimal Care Pathway for Aboriginal and Torres Strait Islander People with Cancer*, which states that an IHW or ILO “*should oversee care to ensure it is culturally appropriate and provide emotional, social and cultural support to patients, their families and carers*” and that it is “*essential that the team includes an expert in providing culturally appropriate care to this population*” [26]. However, previous studies suggest that mainstream tertiary health services rarely have sufficient Indigenous health professionals (including ILOs and IHWs) to meet patient needs [46, 50, 61]. Compared to most other hospitals included in the initial survey of cancer services, where 27% didn’t employ any Indigenous staff and 21% were unsure whether Indigenous staff were employed at their health service [36], the two hospitals in this study employed many Indigenous staff, including in identified Indigenous roles. This likely reflects their high level commitment to Indigenous workforce: both hospitals have active RAPs which make accountable commitments to growing and supporting their Indigenous workforce, as well as specific and measurable targets set for the employment of Indigenous staff.

Most participants described the time between first experiencing symptoms and reaching either the Urban or Regional Cancer Service as “*confusing*” and “*stressful*”, attributed in part to issues navigating the health system. This echoes previous studies, which found that the barriers faced by many Indigenous people in accessing specialist and hospital care are substantial, and include: long wait times for diagnostic tests and appointments with cancer specialists, misdiagnosis by General Practitioners (GPs) and communication difficulties all contributed to delayed diagnosis of cancer for Indigenous people [46, 61]. Although these two services had made some efforts to mitigate the barriers experienced by patients before commencing treatment, such as direct admission to the oncology ward (bypassing the Emergency Department), providing additional pre-admission information to Indigenous patients and early involvement of the ILO acting in an informal care coordination role, the reported patient experiences indicate that there is room for improvement. Both primary and tertiary care providers can do more to prepare Indigenous patients for the experience of admission to a tertiary cancer treatment service, including providing information about where to go, what to bring and likely length of relocation or admission for treatment. Telehealth should be offered for pre-admission consultations and used to improve pre-hospital orientation, especially for regional/remote patients [62]. Formalised patient navigator and cancer care coordinator positions in both primary and tertiary care services have been found to help Indigenous people navigate the health system and facilitate continuity of care [35, 63]. However, cancer care coordination is not available in in all metropolitan hospitals in Australia, let alone in regional hospitals or primary care services, and so patient needs may remain unmet [35]. System-level change is required to improve the links between primary health care and tertiary cancer diagnostic and treatment services, and system level change will have benefits for multiple other conditions that require better integrated care as well [64, 65].

The importance of family involvement in care is a recognised feature of Indigenous culture and has been frequently reported [48, 50, 58, 66, 67], as have the limitations of hospital infrastructure and carer funding to support extended Indigenous families [24, 35, 48, 68]. Consistent with findings in the present study, family involvement in care has been found to improve patients’ mental health, aid communication with health professionals and provide important logistical support [48, 58, 67] and an appropriate physical environment can assist but not replace such social support [24]. The services in this study comply with guidance in the *Optimal Care Pathway for Aboriginal and Torres Strait Islander People with Cancer*, which highlights the importance of “*accommodating and encouraging the inclusion of multiple family and/or community members/ Elders at appointments, including the use of modern technology to facilitate this*” in creating an optimal care environment [26]. Family involvement should be offered to patients systematically and routinely at all stages of their engagement with the health service [48]. As our provider participants stressed, it is important that health services do not make assumptions about Indigenous patients, including desired level of family involvement, without consulting the individual patient [58]. It is also important that health services are aware of the significant stress and burden that often occurs for Indigenous carers and family members, who may have complex health situations of their own and additional caring responsibilities beyond those commonly experienced by non-Indigenous carers [69].

Health services wanting to assess their performance from the perspective of Indigenous patients and family members have multiple tools available. However, a recent study which examined the adequacy of four patient experience measures for Indigenous people found while the tools were “*by no means irrelevant to Indigenous people*”, none of the tools completely captured the critical aspects of cancer care as identified by Indigenous people affected by cancer, with a notable lack of questions around culture and cultural safety [70]. This study calls for the development of patient experience measures that are “*strengths-based, reflect an Indigenous worldview and measure aspects of experience relevant to Indigenous people*” [70]. While not specifically assessing patients’ experiences of care, the Supportive Care Needs Assessment Tool for Indigenous People (SCNAT-IP) tool is an evidence based tool which can be used by cancer services to identify and address the unmet supportive care needs of Indigenous people and thereby improve their experience of cancer care [71]. A study into the feasibility of the tool found that most Indigenous cancer patients liked being asked about their supportive care needs, and that it helped improve patient-clinician communication; helped establish greater rapport between staff and patient; and may detect issues not identified by current care protocols [71]. While not cancer specific, the empirically validated Cultural Safety Survey allows hospitals to measure the cultural safety of their services from the perspective of Indigenous patients and evaluate whether efforts to improve cultural safety are resulting in patients reporting more culturally safe experiences [72].

### Limitations

The original intention was for this study to utilise a mixed methods approach, and to incorporate quantitative data such as patient pathway timeframes to support the qualitative data. It was also intended to explore linkages between cancer services and primary health care, which may have provided additional information on the finding that getting to the cancer service can be challenging. However, delays with this project reduced the capacity and timeframes so that this did not occur.

We interviewed only eight Indigenous people directly affected by cancer, with several factors contributing to this. This is a vulnerable group, so care was taken when approaching participants to ensure that their mental health and physical well-being would not be compromised by participating in this study, this limited the number of participants that could be approached. Furthermore, a number of Indigenous patients who agreed to be interviewed were unavailable on the day of the scheduled interview due to poor-health or recent discharge. The voluntary nature of the study means that patients who were less satisfied with their care may have chosen not to participate, and the study did not include those who had not presented, declined treatment or had disengaged from the health system. While including the views of carers and Indigenous health professionals may have mitigated these limitations somewhat, other Indigenous people affected by cancer may have had different experiences from those reported here. Due to the small sample size and the huge diversity of experience we cannot claim data saturation with this participant group.

During analysis of the staff interviews it was noted that the experiences described began to replicate, suggesting that saturation may have been reached amongst this participant group. We attempted to minimise selection bias of staff by interviewing both Indigenous and non-Indigenous staff from a diverse range of professions, including upper management, clinicians, and support staff, as well as through the use of snowballing.

## Conclusion

This article is significant because it demonstrates that with a culturally appropriate and person-centred approach, involving patients, family members, Indigenous and non-Indigenous staff, it is possible for Indigenous people to have positive experiences of cancer care in mainstream, tertiary health services. Furthermore, while the two cancer services and their affiliated hospitals were selected because prior research suggested that they were particularly high performing in their provision of cancer services for Indigenous people, these two health services are vastly different with respect to rurality, management and patient cohort and therefore represent much of the diversity that exists between health services in Australia. If we are to improve health outcomes for Indigenous people it is vital more cancer services and hospitals follow their lead and make a sustained and ongoing commitment to strengthening the cultural safety of their service. Documents such as the *Optimal Care Pathway for Aboriginal and Torres Strait Islander People with Cancer* [26] and *Australian National Safety and Quality Health Service (NSQHS) Standards User guide for Aboriginal and Torres Strait Islander health* [27] provide important guidance on this. In addition, further Indigenous-guided research is needed to identify and evaluate successful cancer service delivery initiatives for Indigenous Australians. This will enable health services to innovate and advance the delivery of cancer care to improve patient outcomes and experience of care for Indigenous cancer patients and their families.

## Supplementary Information

**Additional file 1.**

**Additional file 2.**

## Data Availability

The datasets generated and/or analysed during the current study are not publicly available due to small participant numbers and protection of confidentiality. Aggregate data is available from the corresponding author on reasonable request.

## Abbreviations

ACCHO: Aboriginal Community Controlled Health Organisation
ACD: Advanced Care Directive
AHPRA: Australian Health Practitioner Regulation Agency
ALO: Aboriginal Liaison Officer, titled Indigenous Liaison Officer (ILO) in some hospitals
CRE: Centre for Research Excellence
DISCOVER-TT: Discovering Indigenous Strategies to improve Cancer Outcomes Via Engagement, Research Translation and Training Centre of Research Excellence
ED: Emergency Department
GP: General Practitioner
IHW: Indigenous Health Worker
ILO: Indigenous Liaison Officer, titled an Aboriginal Liaison Officer (ALO) in some hospitals
NHMRC: National Health and Medical Research Council
NSQHS: National Safety and Quality Health Service
NUM: Nurse Unit Manager
RAP: Reconciliation Action Plan
RN: Registered Nurse
WAAHEC: Western Australian Aboriginal Health Ethics Committee
