# Supplementary material for: “The support has been brilliant”: experiences of Aboriginal and Torres Strait Islander patients attending two high performing cancer services
Source: BMC Health Serv Res. 2021 May 24;21:493. doi: 10.1186/s12913-021-06535-9 (PMC8142293; doi:10.1186/s12913-021-06535-9)
Supplement: Supplementary file 1 — Additional file 1. [file 12913_2021_6535_MOESM1_ESM.pdf]

INNOVATIVE MODELS OF CANCER CARE FOR INDIGENOUS AUSTRALIANS

Interview Guide – Health Professionals

General areas to be discussed:

1. Could you tell me about your role in delivering cancer treatment to Aboriginal and Torres Strait Islander patients? (Specialty, number of years practicing, catchment area, role, etc.)

2. What is the typical treatment pathway for patients requiring treatment? (If appropriate - please describe how that differs from 10 years ago)

3. Please describe how decisions about cancer treatment options are generally made? Are patients involved in the decision-making? How are they involved?

4. What are your views on the quality of the services provided by your health service to Aboriginal and Torres Strait Islander patients? (and compared with other service centres) in terms of the following:

    a. Consults with Aboriginal and Torres Strait Islander stakeholders to establish and run programmes/services

    b. Establishes links with primary care providers and integration/continuity of services

    c. Identifies distinctive care needs of Aboriginal and Torres Strait Islander patients and responds to enable better support, e.g. logistical support, psycho-social support, telehealth, etc.

    d. Assesses improvements in cancer outcomes and quality of life (QOL) for Aboriginal and Torres Strait Islander patients

    e. Provides appropriate information and resources for Aboriginal and Torres Strait Islander people affected by cancer around cancer and cancer care

    f. Engages in primary prevention and Aboriginal and Torres Strait Islander community education around cancer and cancer care.

5. How could your health service improve to better meet the needs of Aboriginal and Torres Strait Islander patients? And what changes could improve cancer services generally - do you have any suggestions?

6. Can you describe any barriers (e.g. financial, logistical, etc.) you are aware of for Aboriginal and Torres Strait Islander people accessing treatment at your health service?

7. Is there anything else you would like to add?
