# Supplementary material for: “The support has been brilliant”: experiences of Aboriginal and Torres Strait Islander patients attending two high performing cancer services
Source: BMC Health Serv Res. 2021 May 24;21:493. doi: 10.1186/s12913-021-06535-9 (PMC8142293; doi:10.1186/s12913-021-06535-9)
Supplement: Supplementary file 2 — Additional file 2. [file 12913_2021_6535_MOESM2_ESM.pdf]

THE UNIVERSITY OF WESTERN AUSTRALIA

Western Australian Centre for Rural Health

INNOVATIVE MODELS OF CANCER CARE FOR INDIGENOUS AUSTRALIANS

Interview Guide – People Affected by Cancer

Topics to be discussed:

1. Please tell me a bit about your cancer.

2. Please describe what cancer treatment you have had since your diagnosis? (When, what type of treatment, location of treatment, duration and how much longer?)

3. What sort of things affected your decision to take up treatment? (health system factors, service-related issues, individual factors including personal and social values, for example shame and embarrassment, gender, autonomy, etc.)

4. Could you tell me a little bit about the reasons why you were treated in [name of health service] rather than another location? Who made the decision? Why?

5. Please describe your experience of the service you received in [health service]. Areas that you may want to elaborate on include access, communication with the team (I.e. doctors, social workers, nurses, other staff) and follow-up.

6. Can you please tell me about the support you received during your treatment and who from? (For example Cancer Council, local support group, family)

7. Did you have any issues getting to and from your appointments and attending treatment? (e.g. to do with driving, travel distance, time and expenses)

8. Can you please tell me about any other issues you experienced? For example availability of suitable information, ability to adhere to treatments and caregiver support?

9. What do you think is the understanding of cancer amongst Aboriginal and Torres Strait Islander people? What are their beliefs about cancer and cancer treatment? What/who do they trust? What are their expectations?

10. What issues do you think are particularly important in caring for Aboriginal and Torres Strait Islander patients, often remote from cancer treatment centres (living environments, transport, mobility, and social support)?

11. In your opinion how could services be improved to better meet the needs of Aboriginal and Torres Strait Islander cancer patients? Do you have any suggestions?

12. Are there any comments you would like to make about your access to cancer treatment services as an Aboriginal and Torres Strait Islander person? This could be about the barriers or facilitators to care and

the support that you experienced. What were the things that worked well, and where do you feel the system could be improved?

13. If appropriate, discuss end of life care considerations and needs.

14. Is there anything else that you would like to say about your treatment for cancer?
